# Supplementary material for: Modulation of Dietary Choline Uptake in a Mouse Model of Acid Sphingomyelinase Deficiency
Source: Int J Mol Sci. 2023 Jun 5;24(11):9756. doi: 10.3390/ijms24119756 (PMC10253472; doi:10.3390/ijms24119756)
Supplement: Supplementary file 1 [file ijms-24-09756-s001.zip › ijms-2357685-supplementary.pdf]

International Journal of
Molecular Sciences

Article

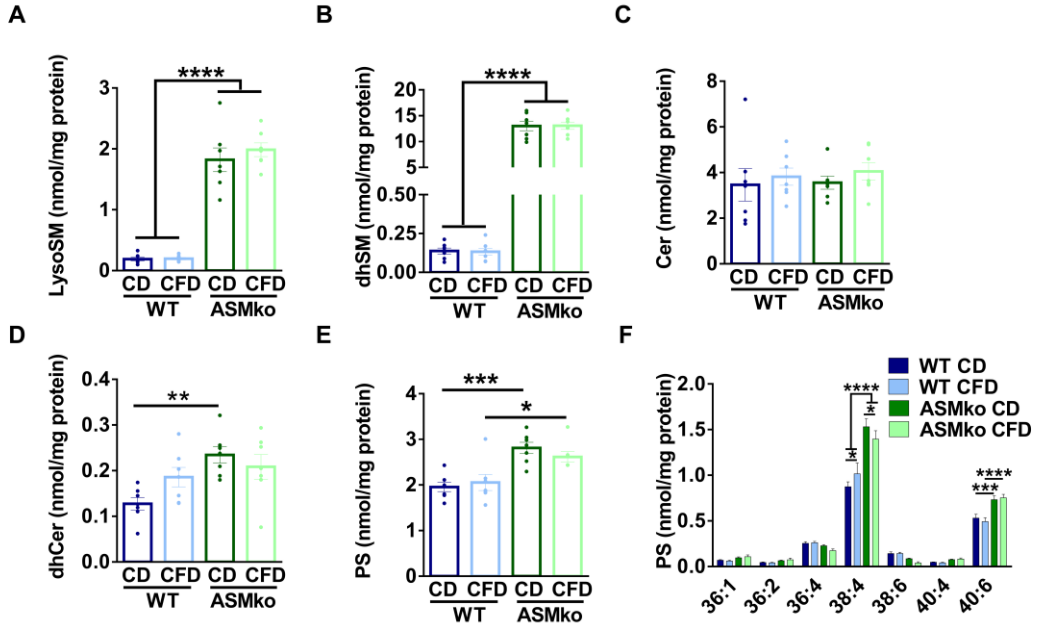

Figure S1. Choline deficiency effects on the liver levels of sphingolipids and phosphatidylserine. A. Graphs show mean ± SEM of total LysoSM levels expressed as nmol/mg protein in extracts from liver of wt and ASMko mice fed a control diet or a choline-free diet (n=7; *****p*<0.0001). B. Graphs show mean ± SEM of total dhSM levels expressed as nmol/mg protein in extracts from liver of wt and ASMko mice fed a control diet or a choline-free diet (n=7; *****p*<0.0001). C. Graphs show mean ± SEM of total Cer levels expressed as nmol/mg protein in extracts from liver of wt and ASMko mice fed a control diet or a choline-free diet (n=7). D. Graphs show mean ± SEM of total dhCer levels expressed as nmol/mg protein in extracts from liver of wt and ASMko mice fed a control diet or a choline-free diet (n=7; ***p*<0.01). E. Graphs show mean ± SEM of total PS levels expressed as nmol/mg protein in extracts from liver of wt and ASMko mice fed a control diet or a choline-free diet (n=7; **p*<0.05; ****p*<0.001). F. Graphs show mean ± SEM of the indicated PS levels expressed as nmol/mg protein in extracts from liver of wt and ASMko mice fed a control diet or a choline-free diet (n=7; **p*<0.05; ****p*<0.001; *****p*<0.0001).

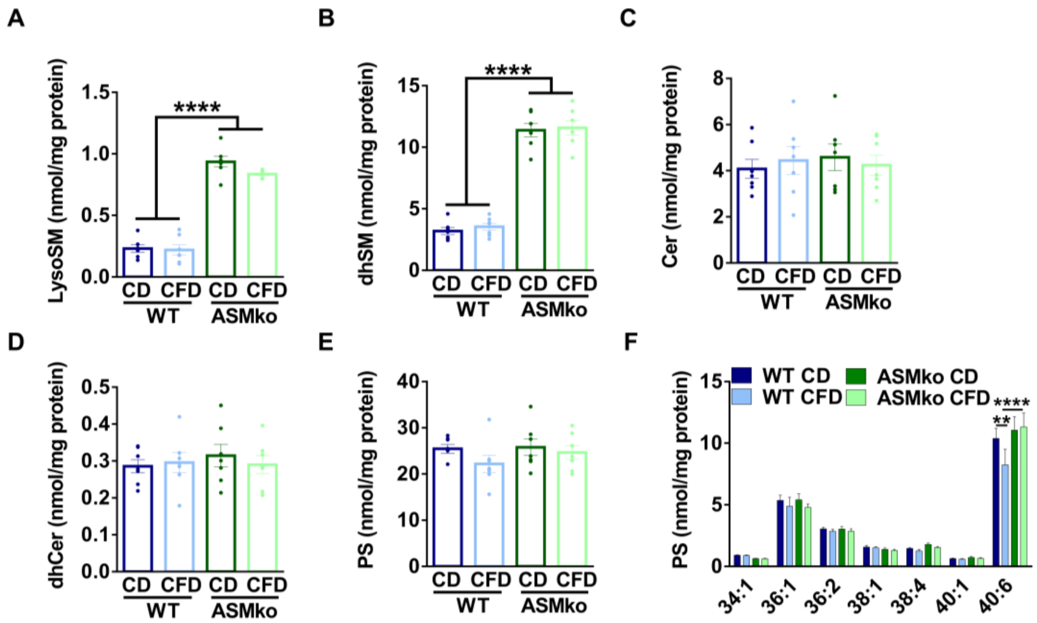

Figure S2. Choline deficiency effects on the brain levels of sphingolipids and phosphatidylserine. A. Graphs show mean ± SEM of total LysoSM levels expressed as nmol/mg protein in extracts from cerebellum of wt and ASMko mice fed a control diet or a choline-free diet (n=7; *****p*<0.0001). B. Graphs show mean ± SEM of total dhSM levels expressed as nmol/mg protein in extracts from cerebellum of wt and ASMko mice fed a control diet or a choline-free diet (n=7; *****p*<0.0001). C. Graphs show mean ± SEM of total Cer levels expressed as nmol/mg protein in extracts from cerebellum of wt and ASMko mice fed a control diet or a choline-free diet (n=7). D. Graphs show mean ± SEM of total dhCer levels expressed as nmol/mg protein in extracts from cerebellum of wt and ASMko mice fed a control diet or a choline-free diet (n=7; ***p*<0.01). E. Graphs show mean ± SEM of total PS levels expressed as nmol/mg protein in extracts from cerebellum of wt and ASMko mice fed a control diet or a choline-free diet (n=7; **p*<0.05; ****p*<0.001). F. Graphs show mean ± SEM of the indicated PS levels expressed as nmol/mg protein in extracts from cerebellum of wt and ASMko mice fed a control diet or a choline-free diet (n=7; **p*<0.05; ****p*<0.001; *****p*<0.0001).
